# Prediction of Surface Roughness considering Cutting Parameters and Humidity Condition in End Milling of Polyamide Materials

**DOI:** 10.1155/2018/5850432

**Published:** 2018-06-28

**Authors:** Mustafa Bozdemir

**Affiliations:** Department of Mechanical and Metal Technologies, Kırıkkale Vocational High School, Kırıkkale University, Kırıkkale, Turkey

## Abstract

To know the impact of processing parameters of PA6G under different humidity conditions is important as it is vulnerable to humidity up to 7 %. This study investigated the effect of cutting parameters to surface roughness quality in wet and dry conditions. Artificial Neural Network (ANN) modeling is also developed with the obtained results from the experiments. Humidity condition, tool type, cutting speed, cutting rate, and depth of cutting parameters were used as input and average surface roughness value were used as output of the ANN model. Testing results showed that ANN can be used for prediction of average surface roughness.

## 1. Introduction

Usage of engineering plastics has been constantly increasing due to its characteristics of lightness, cheapness, and strength. Today, it has a wide range of usage nearly in all the fields of industry. There are different types and characteristics of engineering plastics. Polyamides (especially cast polyamide or Castamide as industrial name) are one of these most widespread plastic types [1]. PA6G has superior properties over many metals by being cheaper, easily processed, light, high-resistant, and abrasion-resistant. Many studies have been carried out for different characteristics of polyamides (friction condition [2], wear properties [3], thermal properties [4], machinability [5], etc.). In this study, machinability is concerned.

There are various studies to the aim of detecting the relationship between surface roughness and cutting conditions [6–19]. Davim has studied the difference between processing conditions of turn bench and surface roughness formation of glass fiber reinforced and nonreinforced PA66 polyamide materials [6]. Bozdemir carried out experiments on PA6G materials having the characteristic of dehumidification up to 7%, resulting in that average surface roughness value changes although process conditions have not changed for dehumidified PA6G material [7].

Ho et.al. combined ANFIS with hybrid Taguchi-genetic learning algorithm for prediction of surface roughness in end milling process [8]. Dong and Wang proposed an improved approach to model surface roughness with adaptive network-based fuzzy inference system (ANFIS) and leave-one-out cross-validation (LOO-CV) approach [9]. Zain et.al. studied the prediction of surface roughness performance measure in end milling machining operation by using regression and ANN modeling techniques [10, 11]. Fung et.al. used nonlinear stochastic exogenous autoregressive moving average model neural network identification method to model the workpiece radial and longitudinal errors of a lathe turning machine [12]. Correa et al. investigated efficacy of two different machine learning classification methods, Bayesian networks and artificial neural networks, for predicting surface roughness in high-speed machining [13]. Asıltürk and Çunkaş used artificial neural networks and multiple regression approaches to model the surface roughness of AISI 1040 steel [14].

The observation of surface roughness values by using optimization methods and predictions based on artificial mind techniques can also be made [15–19]. Yilmaz et al. studied the predicting of the surface roughness by means of neural network approach on machining of an extruded PA6G cast polyamide. They used 2 inputs called spindle speed and feed rate in the model. Output of the model is surface roughness (Ra). They applied gradient descent method to optimize the weight parameters of neuron connections [15]. Bozdemir used ANN to determine the effects of materials, cutting speed (V_c_), feed rate (f), diameter of cutting equipment, and depth of cut (a_p_) on average surface roughness. The experimental results were used to train and test. 100 experimental results were used, from the total of 120, as data sets to train the network, while 20 results were used as test data [16]. Bozdemir and Aykut used more data in another study and studied LM, SCG, and CGP algorithms and found LM algorithm to be the best [17].

In this study, different ANN models were developed with the purpose of guessing unachievable gap values and studying carefully the creation of Ra values in the experimental results. ANN is used to determine the effects of cutting conditions (cutting speed, feed rate, diameter of cutting equipment, and depth of cut) besides tool type and humidity conditions on average surface roughness. For each tool type and humidity condition 192 experimental data sets are used in modeling. The results of the model indicate a relatively good agreement between the predicted values and the experimented ones.

## 2. Experimental Setup

**Figure 1:**
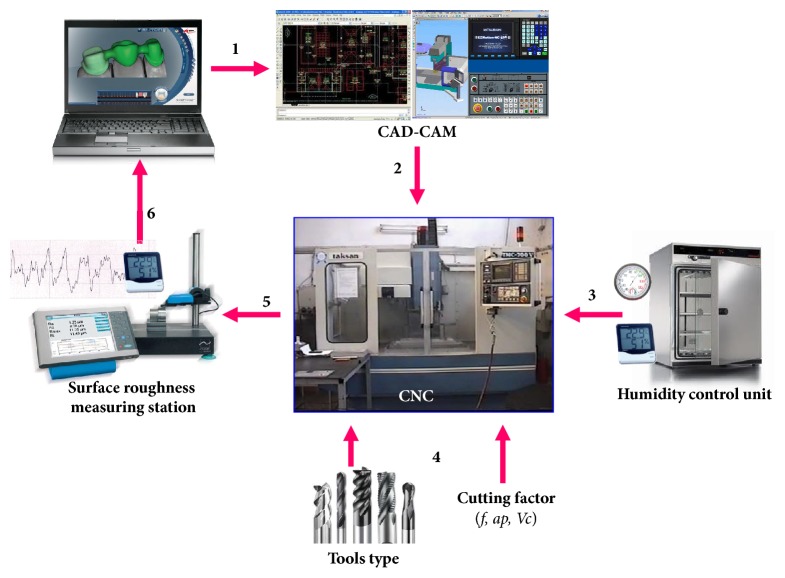
Schematic picture of experiment setting.

PA6G material in 46 mm plates that is used in the experiments is supplied from Polimersan firm. PA6G obtained in plates are cut in dimensions of 112x82x46 mm. and they are kept in humid and dry place. Humid and dry samples are processed in TMC500 CNC vertical machining center. Cycle of bench can be adjusted between 60 and 6000 cycles/min. Carbide and HSS cutting tools are used in experiment. Detailed cutting conditions are given in Table 1. Schematic picture of experiment setting is depicted in Figure 1.

**Figure 2:**
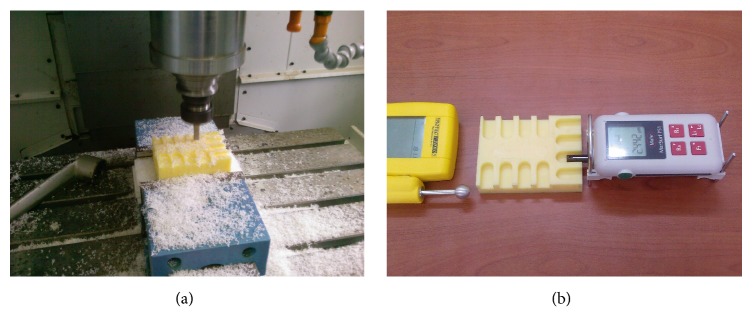
(a) Work piece under process. (b) Measuring machined surface.

For the measurement of roughness, MarSurf PS1 portable surface roughness measurement equipment is used. Measurement needle has the measurement diameter of 2 *μ*m and pressure force is averagely 0.7 mN. Measurement scanning length is adjusted as 5.6 mm. In the measurement of environment and material humidity, rates Trotec T2000S trademark measurement equipment and electronic assay balance are used. Machining of workpiece and measuring machined surface is shown in Figure 2.

## 3. ANN as Prediction Methodology

ANNs offer a computational approach that is quite different from conventional digital computation. Digital computers operate sequentially and can do arithmetic computation extremely fast. Biological neurons in the human brain are extremely slow devices and are capable of performing a tremendous amount of computation tasks necessary to do everyday complex tasks, commonsense reasoning, and dealing with fuzzy situations. The underlining reason is that, unlike a conventional computer, the brain contains a huge number of neurons, information processing elements of the biological nervous system, acting in parallel. ANNs are thus a parallel, distributed information processing structure consisting of processing elements interconnected via unidirectional signal channels called connection weights. Though modeled after biological neurons, ANNs are much simplified and bear only superficial resemblance [20].

**Figure 3:**
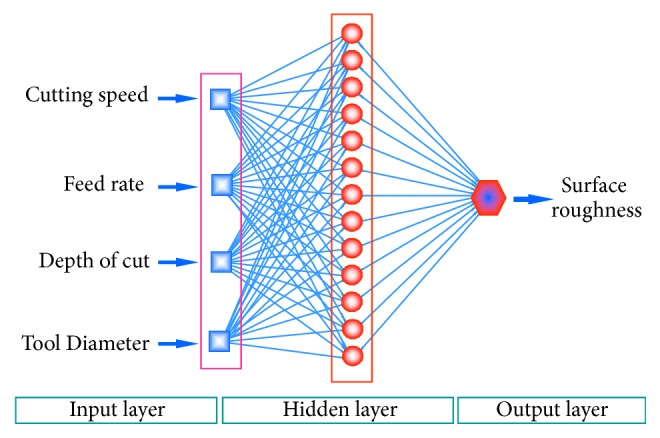
General Representation of an ANN model.

Artificial neural networks vary according to connections between neurons (single layer and multilayer) and function used in neurons (linear, sigmoid, Gaussian, etc.).  Figure 3 shows a general representation of an ANN model. In this study, different ANNs are trained and the one giving best predictions is chosen as the ANN model. Exhaustive Prune, Radial Basis Function Network (RBFN), and Multilayer type ANNs are used. Multilayer type ANNs are tested for the conditions having different number of hidden layers (up to 3) and different number of neurons (up to 20) when it is available.

Experiments were conducted and a data set was obtained containing 192 sets of input parameters for each humidity and tool condition and the corresponding output parameter. This data set was used for training and testing the neural network model. The set was then divided into two parts. One part contained 129 data points and was used for training the network. Another part contained 63 data points and was used for testing the network. The data set used for testing the network was chosen randomly from the experimental data set. Some statistical methods, R^2^, RMSE, and MAPE values have been used for comparison. Some selected sample data sets used for training and testing the network are shown in Table 2.

## 4. Results and Discussion

All ANN types are tested and the ones giving best correlation are used in the cases. This section is divided into four cases according to humidity and to condition and results for each case are given under appropriate part. Number of input and output parameters is kept constant as 4 and one during modeling. It is seen that Exhaustive Prune and RBFN are the ANNs giving more accurate results.

Considering inputs as numerical values eases the modeling process because ANN finds out relations between inputs and outputs by using formulas. On the other hand, some recent techniques (C&R Tree, CHAID, etc.) focus on the classification of the data and they can handle numerical and alpha-numerical characters as inputs more effectively. In this study, different ANN models are tested using whole data (including humidity condition and tool type) and results showed weak correlation (under 60%) between predicted and experimental data. So, the results are given at different sections in the following paragraphs.

Another result that should be mentioned is the efficacy of multilayer ANNs. Generally they outperformed the single layer ones as predicted. These models have the advantage of having much more flexibility in defining constants in relation formulas of neurons. As a consequence, they have better adaptability in defining the relation between inputs and outputs.

Number of neurons is also another effective parameter in finding best correlation. Having more neurons can increase efficacy of the model up to certain values as can be seen in literature [16, 17]. Even though Bozdemir has selected one layer, he could manage to get correlation as much as ours by means of using 13 neurons while we have only 2-3 neurons in each layer. On the other hand, Yilmaz et.al. managed to build an effective ANN model by using second hidden layer having 3-4 neurons [15].

### 4.1. Wet Material Machined with Carbide Tool

**Figure 4:**
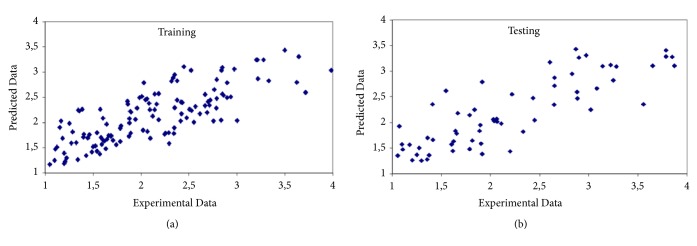
Predicted versus experimental values for first case. (a) Training. (b) Testing.

Exhaustive Prune is the best choice for this case. The model constructed has 82.4% correlation and 0.322 relative error. Afour layer network, with one input layer, two hidden layers, and one output layer, was formed for the present case. The first hidden layer has 3 neurons and second hidden layer has 2 neurons.  Table 3 gives the statistical evaluation of prediction and Figure 4 shows the predicted values versus experimental values for training and testing.

### 4.2. Dry Material Machined with Carbide Tool

**Figure 5:**
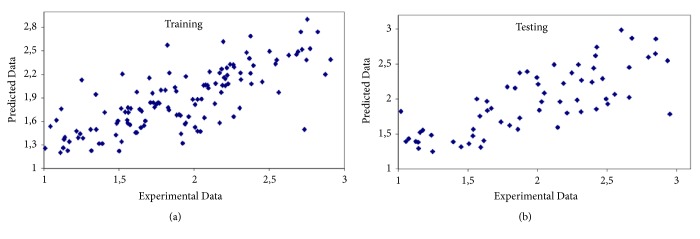
Predicted versus experimental values for second case. (a) Training. (b) Testing.

Exhaustive Prune is the best choice for this case. The model constructed has % 76.5 correlations and 0.421 relative error. A four-layer network, with one input layer, two hidden layers, and one output layer, was formed for the present case. The first hidden layer has 5 neurons and second hidden layer has 4 neurons.  Table 4 gives the statistical evaluation of prediction and Figure 5 shows the predicted values versus experimental values for training and testing.

### 4.3. Wet Material Machined with HSS Tool

**Figure 6:**
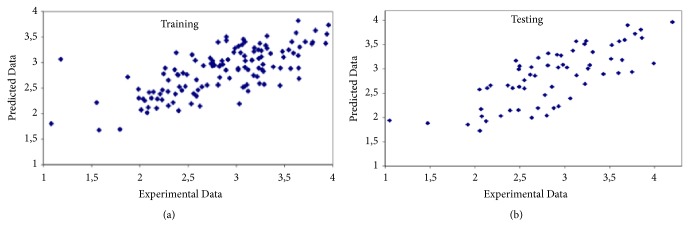
Predicted versus experimental values for third case. (a) Training. (b) Testing.

RBFN is the best choice for this case. The model constructed has % 76.3 correlations and 0.444 relative error. A three-layer network, with one input layer, two hidden layers, and one output layer, was formed for the present case. The first hidden layer has 20 neurons.  Table 5 gives the statistical evaluation of prediction and Figure 6 shows the predicted values versus experimental values for training and testing.

### 4.4. Dry Material Machined with HSS Tool

**Figure 7:**
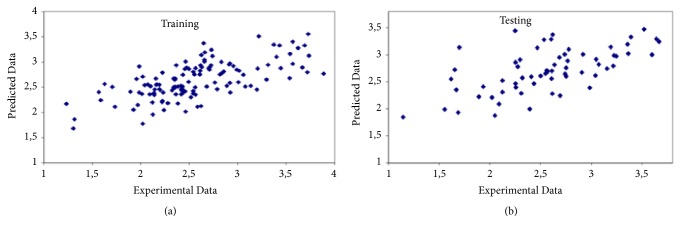
Predicted versus experimental values for fourth case. (a) Training. (b) Testing.

RBFN is the best choice for this case. The model constructed has % 76.3 correlations and 0.444 relative error. A three-layer network, with one input layer, two hidden layers, and one output layer, was formed for the present case. The first hidden layer has 20 neurons.  Table 6 gives the statistical evaluation of prediction and Figure 7 shows the predicted values versus experimental values for training and testing.

## 5. Conclusion

The aim of this paper is to investigate the effects of humidity, tool type, cutting speed, feed rate, diameter of cutting equipment, and depth of cut on average surface roughness using the neural networks. Analyses of ANN for different materials (dry and humid PA6G) having different cutting conditions are performed in average surface roughness.

This paper introduces ANN's technique for modeling the average surface roughness. 129 results were used as data sets to train the network, while 63 results were used as test data from the total experimental results of each case. Different algorithms were studied and not a unique perfect technique is determined but the best results were obtained from exhaustive prune and RBFN algorithms. For testing data, correlation values have changed from %62.7 to %82.4. This difference could occur because of the change in importance of factors affecting the process. To train with more data could also improve the correlation. On the other hand, these ANN predicted results can be considered within acceptable limits. The results show good agreement between predicted and measured values.

## Figures and Tables

**Table 1:** Cutting conditions.

**Cutting factor**	**Unit**	**Data levels**
Feed rate, [f]	mm/min.	100 - 125 - 150 -175
Depth of cut, [a_p_]	mm	1 - 1.5 - 2 - 2.5
Cutting speed, [V_c_]	m/min.	100 - 120 - 140 - 160
Tool diameter, [C]	mm	10 - 14 - 18
Tool type, [T]	-	HSS and Carbide
Humidity, [H]	-	Dry and Wet

**Table 2:** Sample data sets used for training and testing.

**Input parameters **				**Output parameter**			
				**HSS Tool**	**HSS Tool **	**Carbide **	**Carbide **
Depth of cut	Cutting speed	Diameter of	Feed rate	**(Dry) **	**(Wet)**	**Tool (Dry)**	**Tool (Wet)**
(mm)	(m/min)	cutting (mm)	(mm/min)	**Ra(** **µ** **mm)**	**Ra(** **µ** **mm)**	**Ra (** **µ** **mm)**	**Ra(** **µ** **mm)**
1	100	⌀10	100	**0,927**	**2,184**	**2,919**	**3,045**
1	100	⌀14	100	**1,129**	**1,571**	**2,341**	**3,283**
1	120	⌀10	125	**1,574**	**3,330**	**2,465**	**3,355**
1	120	⌀18	125	**2,005**	**2,028**	**1,936**	**2,550**
1	140	⌀10	125	**2,188**	**2,894**	**2,342**	**2,730**
1	140	⌀18	150	**2,247**	**3,023**	**2,752**	**3,750**
1	160	⌀14	100	**1,260**	**1,582**	**1,928**	**2,052**
1	160	⌀18	175	**2,935**	**2,650**	**2,754**	**2,958**
1,5	100	⌀10	100	**1,158**	**2,652**	**2,253**	**2,490**
1,5	100	⌀18	100	**1,404**	**1,685**	**2,923**	**2,246**
1,5	120	⌀10	150	**2,494**	**3,281**	**2,620**	**3,236**
1,5	120	⌀18	150	**2,199**	**2,473**	**2,075**	**2,378**
1,5	140	⌀10	125	**2,266**	**2,027**	**2,162**	**3,279**
1,5	140	⌀18	125	**2,090**	**2,340**	**2,100**	**2,390**
1,5	160	⌀10	125	**1,541**	**1,598**	**2,280**	**2,833**
1,5	160	⌀14	175	**1,946**	**2,045**	**2,500**	**2,773**
1,5	160	⌀18	175	**2,239**	**2,139**	**3,209**	**3,930**
2	100	⌀10	100	**3,225**	**2,972**	**3,520**	**3,932**
2	100	⌀10	175	**3,952**	**3,787**	**4,520**	**4,860**
2	140	⌀10	125	**1,874**	**1,873**	**2,901**	**3,242**
2	140	⌀14	125	**1,350**	**1,182**	**2,036**	**2,388**
2	160	⌀10	175	**2,719**	**1,816**	**3,369**	**4,234**
2	160	⌀14	175	**1,738**	**1,429**	**3,287**	**3,647**
2	160	⌀18	175	**2,170**	**1,678**	**3,015**	**3,807**
2,5	100	⌀10	125	**2,310**	**1,354**	**2,629**	**3,073**
2,5	100	⌀18	150	**1,565**	**1,889**	**2,637**	**2,898**
2,5	140	⌀10	125	**1,416**	**1,540**	**2,347**	**2,794**
2,5	140	⌀14	100	**1,176**	**0,918**	**2,393**	**2,555**
2,5	140	⌀18	150	**1,880**	**1,506**	**2,567**	**3,211**
2,5	140	⌀18	175	**1,973**	**1,732**	**2,865**	**3,490**
2,5	160	⌀10	100	**1,122**	**1,195**	**1,864**	**2,143**
2,5	160	⌀14	175	**1,823**	**1,553**	**2,897**	**3,051**
2,5	160	⌀18	175	**2,836**	**2,283**	**3,168**	**4,181**

**Table 3:** Statistical evaluation of prediction for first case.

	Training	Testing
Minimum Error	0.0056	0.0014
Maximum Error	1.113	1.21
Mean Absolute Error	0.324	0.354
Correlation	0.784	0.824

**Table 4:** Statistical evaluation of prediction for second case.

	Training	Testing
Minimum Error	0.0047	0.0018
Maximum Error	1.241	1.169
Mean Absolute Error	0.228	0.28
Correlation	0.787	0.765

**Table 5:** Statistical evaluation of prediction for third case.

	Training	Testing
Minimum Error	0.013	0.011
Maximum Error	1.884	0.889
Mean Absolute Error	0.314	0.346
Correlation	0.715	0.763

**Table 6:** Statistical evaluation of prediction for fourth case.

	Training	Testing
Minimum Error	0.0038	0.0042
Maximum Error	1.117	1.435
Mean Absolute Error	0.334	0.351
Correlation	0.666	0.627

## Data Availability

The [average surface roughness values] data used to support the findings of this study have been deposited in the “The Effects of Humidity on Cast PA6G during Turning and Milling Machining, Advances in Materials Science and Engineering Volume 2017 (2017), Article ID 5408691” repository (https://doi.org/10.1155/2017/5408691).
